# Atherogenic Dyslipidemia and Its Association with *FTO* Gene Polymorphisms in Working Perimenopausal Women

**DOI:** 10.3390/ijms262210915

**Published:** 2025-11-11

**Authors:** Astrid Lorena Urbano Cano, Rosa Elvira Álvarez Rosero, Yamil Liscano

**Affiliations:** 1 Grupo de Investigación en Salud Integral (GISI), Facultad de Salud, Universidad Santiago de Cali, Cali 760035, Colombia; 2 Grupo de Investigación en Genética Humana Aplicada (GIGHA), Departamento de Ciencias Fisiológicas, Universidad del Cauca, Popayan 190003, Colombia

**Keywords:** atherogenic dyslipidemia, *FTO*, genetic polymorphisms

## Abstract

Atherogenic dyslipidemia (AD) is a high-risk phenotype for cardiovascular disease, characterized by elevated triglycerides, increased small dense low-density lipoprotein cholesterol (sdLDL-C), and frequently coexisting hypertension. Although *FTO* gene variants have been implicated in lipid dysregulation, their role in AD among Latin American women remains poorly defined. We conducted a case–control study in 219 working perimenopausal women (97 AD cases and 122 controls). Sociodemographic, clinical, and biochemical variables were assessed. Three *FTO* SNPs (*rs9939609*, *rs9940128*, and *rs8050136*) were genotyped. Associations were evaluated using logistic regression models adjusted for age and BMI, with gene–environment interactions tested for smoking. Linkage disequilibrium (LD) and haplotype analyses were also performed. Women with AD exhibited significantly higher triglycerides, LDL-C, and sdLDL-C, along with increased hypertension prevalence, but no differences in BMI or glycemia. Multivariable models identified LDL-C (aOR ≈ 8), triglycerides, sdLDL-C, and systolic blood pressure as the strongest determinants of AD. The *rs8050136* AA genotype was associated with a fourfold higher risk (aOR = 4.12; 95% CI: 1.49–11.95, *p* = 0.007). Smoking independently doubled AD risk (aOR = 2.33) and amplified the effect of *rs8050136*. Adjusted haplotype analysis revealed that the A-A-A (aOR = 5.33; 95% CI: 1.42–20.00) and A-G-A combinations (aOR = 2.54; 95% CI: 1.01–6.38) were significantly associated with AD. *FTO* polymorphisms, particularly *rs8050136* and the A-A-A and A-G-A haplotypes, contribute independently and supra-additively to AD risk. The observed gene–environment interaction with smoking emphasizes the multifactorial nature of AD and supports genotype-based risk stratification and targeted preventive strategies in precision cardiovascular medicine.

## 1. Introduction

Cardiovascular Disease (CVD) remains the leading cause of morbidity and mortality worldwide, representing a major biomedical challenge at the pathophysiological level and reflecting the complex interplay between genetic and environmental factors [1]. In this context, one of the most clinically relevant metabolic factors associated with CVD is atherogenic dyslipidemia (AD), a lipid profile disorder characterized by elevated triglyceride levels, reduced concentrations of high-density lipoprotein cholesterol (HDL-C), and an increased proportion of small, dense low-density lipoprotein cholesterol (sdLDL-C), with or without concomitant elevation in low-density lipoprotein cholesterol (LDL-C) [2]. AD is regarded as a major determinant in the onset and progression of atherosclerosis, as it promotes plaque formation and advancement through mechanisms such as endothelial dysfunction, vascular inflammatory responses, and lipid-driven immune activation, thereby substantially increasing both cardiovascular and atherothrombotic risk [3]. This phenotype functions not only as a clinical marker but also as evidence of a deeper disruption in the regulation of bioactive lipid metabolism, including oxidized lipoproteins, ceramides, and sphingolipids, whose signaling activity plays a critical role in oxidative stress and cellular apoptosis [4,5]. The study of how bioactive molecules interact with complex lipid assemblies, such as cell membranes, is a critical area of research that informs the development of novel therapeutic compounds [6,7] and antimicrobial agents [8,9]. In Latin America, the prevalence of AD ranges from 18% to 24%, with studies indicating that contributing factors include unhealthy lifestyles, genetic predispositions, and epigenetic modifications [10]. In addition, a recent genetic and biochemical evaluation of an urban cohort comprising 304 individuals demonstrated that participants with dyslipidemia exhibited significantly elevated total cholesterol and very-low-density lipoprotein (VLDL) levels, concomitant reductions in HDL, and an increased Castelli risk index II [11]. Collectively, these findings underscore the complex interplay between lipid abnormalities and cardiovascular risk, highlighting the urgent need for targeted public health strategies and personalized clinical management approaches aimed at populations at a heightened risk.

Furthermore, during the perimenopausal transition, some women experience significant alterations in their lipid profile, contributing to the development of AD [12]. This disturbance is particularly relevant, as it can occur silently and exacerbate cardiometabolic risk. Although much of the research has focused on dyslipidemia in the general population, certain subgroups, such as actively employed perimenopausal women, warrant special attention due to their unique physiological and socio-environmental characteristics [13]. While perimenopausal women are generally considered to have a relatively lower cardiovascular risk than postmenopausal women, this is primarily attributed to the cardioprotective effects of endogenous estrogens [14]. The increasing incidence of AD among actively employed perimenopausal women may reflect the interaction between a significant genetic predisposition and environmental factors related to occupational setting and lifestyle. Recent studies suggest that occupational stress, irregular eating patterns, and reduced physical activity contribute to critical metabolic alterations in the lipid profile [13,15,16].

However, comparative evidence between women with and without formal employment is still lacking; thus, it cannot be ruled out that environmental and social factors inherent to active employment may play a more decisive role than genetic predisposition in the observed increase in AD.

Given these observations, we next sought to elucidate the role of *FTO* in lipid metabolism, as this RNA demethylase is known to modulate gene expression through epitranscriptomic mechanisms. Notably, accumulating evidence indicates that altered RNA methylation status is intimately linked to obesity and downstream metabolic disorders [17,18]. Variants of the *FTO* gene have been associated with increased fat mass and obesity. *FTO*, expressed in the brain, liver, and adipose tissue, plays a key role in regulating lipid metabolism. Its hyperactivity promotes fat accumulation, whereas its inhibition alleviates obesity in murine models [19,20]. In this context, variants of the *FTO* gene (fat mass and obesity-associated gene) have gained considerable attention over recent decades due to their involvement in body weight regulation, adiposity, and insulin resistance, all factors closely linked to AD [21]. However, information regarding the specific role of *FTO* polymorphisms in actively employed perimenopausal women remains limited, despite this group potentially being particularly susceptible to complex cardiometabolic risks. Recent studies have suggested that *FTO* variants may modulate lipid responses to dietary and hormonal factors, which are especially relevant in women approaching menopause, whose physiology is undergoing continuous changes [22]. Furthermore, the interaction between *FTO* polymorphisms and declining estrogen levels during this period may exacerbate adverse effects on lipid metabolism, promoting a more pronounced atherogenic profile [23]. In particular, the intronic variants *rs9939609* (T > A), *rs9940128* (G > A), and *rs8050136* (C > A) have been associated with upregulated *FTO* expression, increased adipose tissue accumulation, and significant alterations in lipid profiles.

The selection of these variants is justified because they represent the main haplotype block within intron 1 of the *FTO* gene associated with lipid and metabolic disorders. Additionally, these variants have been studied in different populations, where their frequency is relatively high among Europeans, Latin Americans, and Asians (*rs9939609* (A): ~0.40 Europeans, ~0.30 Latin Americans, ~0.15 Asians; *rs8050136* (A): ~0.39 Europeans, ~0.25 Latin Americans, ~0.16 Asians strong LD with *rs9939609* and *rs9940128* (G): ~0.32-0.36 in most populations). Consequently, the relevance of analyzing these variants in the context of AD and specifically in this group of perimenopausal women is recognized. In addition to the previously mentioned variants, three others (*rs1421085*, *rs9930506*, and *rs3751812*) are also recognized as being associated with altered lipid profiles, although their effect appears to be related to the environmental and genetic contexts of the different populations.

Notably, the A allele of *rs9939609* has been correlated with elevated triglyceride levels and reduced HDL-C in adult female populations [20,24,25]. In perimenopausal women, the presence of these genetic polymorphisms may exacerbate metabolic alterations associated with the progressive decline in ovarian function, thereby facilitating the onset of AD even in the absence of obesity [26]. This effect is particularly concerning given that it may remain clinically silent, predisposing individuals to heightened cardiometabolic risk. Furthermore, the occupational environment can serve as a significant modulatory factor, influencing the expression of adverse metabolic phenotypes in carriers of high-risk genetic variants [23]. Workplace stress, physical activity patterns, and exposure to environmental factors collectively interact with underlying genetic susceptibilities, amplifying their impact on lipid metabolism and cardiovascular health.

Such an integrative understanding of gene–environment interactions is essential for the development of personalized intervention strategies that combine genetic, clinical, and occupational data. By incorporating these multidimensional risk factors, predictive and preventive approaches can be optimized to mitigate the onset and progression of AD in this vulnerable population [20]. Ultimately, this framework supports the advancement of precision medicine, enabling more targeted monitoring, lifestyle modification, and pharmacological interventions tailored to individual genetic and environmental profiles. Currently, no studies in Colombia have specifically investigated the association between AD and *FTO* gene polymorphisms within the context of occupational exposures. Nevertheless, investigations conducted in the general population of the Colombian Caribbean region, as well as among indigenous communities, have documented significant associations between various Single Nucleotide Polymorphisms (SNPs) and perturbations in lipid and glucose homeostasis [27,28]. These findings imply that, while the modulatory impact of environmental factors on *FTO* remains unexplored, genetic variants may substantially influence the expression of adverse metabolic phenotypes across diverse population subgroups.

By clarifying how these genetic variants influence the lipid profile, a greater understanding of the role of the *FTO* gene can be achieved, along with the identification of potential biomarkers for the early detection and personalized prevention of these conditions [29], holding a similar promise for cardiometabolic risk stratification. In this context, perimenopausal women with formal employment represent a priority group for AD prevention strategies, given their growing participation in the workforce and increased exposure to occupational and metabolic risk factors. This study aims to examine the association between AD and *FTO* gene polymorphisms in employed women, with the purpose of elucidating the genetic and metabolic mechanisms underlying cardiovascular risk.

## 2. Results

### 2.1. Patient Clinical and Sociodemographic Characteristics

A total of 219 women were evaluated, divided into a non-atherogenic dyslipidemia (non-AD) group (*n* = 122) and an AD group (*n* = 97). The overall median age was 48 years (IQR = 13), with no statistically significant difference between groups. The participants’ clinical and biochemical characteristics are detailed in Table 1. Cases presented a markedly less favorable cardiovascular profile, with significantly higher median systolic blood pressure compared with controls (143 mmHg [IQR = 120–170] vs. 122 mmHg [IQR = 110–140]; *p* = 0.011), while diastolic blood pressure also tended to be higher, although the difference did not reach statistical significance (92 mmHg [IQR = 70–99] vs. 87 mmHg [IQR = 70–90]; *p* = 0.060). The prevalence of hypertension was significantly greater in the AD group (81.4% vs. 68.9%; *p* = 0.034), underscoring the strong association between elevated blood pressure and dyslipidemia status. Regarding metabolic parameters, serum triglyceride concentrations (172 mg/dL [IQR = 140–177] vs. 134 mg/dL [IQR = 105–150]; *p* < 0.001), LDL-C (138 mg/dL [IQR = 117–150] vs. 118 mg/dL [IQR = 99–115]; *p* < 0.001), and small dense LDL-C (42 mg/dL [IQR = 32–55] vs. 28 mg/dL [IQR = 20–30]; *p* < 0.001) were significantly elevated in cases, whereas no meaningful differences were observed for total cholesterol, HDL-C, glycemia, or VLDL. Anthropometric variables, including body mass index and waist circumference, were similar across groups, suggesting that obesity-related measures alone do not explain the observed metabolic alterations.

Beyond clinical and metabolic factors, sociodemographic comparisons revealed that marital status differed substantially, with fewer cases being in a relationship compared with controls (39.2% vs. 63.1%; *p* < 0.001), while no significant differences were observed in diabetes prevalence, area of residence, educational attainment, income, or smoking status. Collectively, these findings emphasize the pivotal role of blood pressure and atherogenic lipid fractions as central correlates of AD in this population, while also pointing to the limited explanatory value of anthropometric and sociodemographic variables.

In the binary logistic regression model, higher systolic and diastolic blood pressure were identified as strong independent predictors of AD, with diastolic blood pressure showing an odds ratio (OR) of 4.57. Triglycerides, LDL, sdLDL-C, and VLDL were also significantly associated with increased AD risk, further reinforcing the central role of atherogenic lipid profile abnormalities in the development of this condition. In addition, the *FTO*_*rs9939609* and *FTO*_*rs8050136* genetic variants were associated with a markedly elevated risk, each presenting OR values of approximately 3 or higher. Educational level emerged as a significant protective factor, suggesting a possible socioeconomic or behavioral influence. In contrast, fasting glucose, BMI, HDL, and waist circumference did not reach statistical significance in this adjusted model (*p* > 0.05) (Table 2).

### 2.2. Multivariable Logistic Regression Analysis

The multivariable logistic regression model incorporated a comprehensive set of clinical and biochemical variables, including systolic and diastolic blood pressure, triglycerides, LDL-C, total cholesterol, HDL-C, sdLDL-C, VLDL, glycemia, BMI, age and educational level. The model summary statistics revealed a −2 log-likelihood of 218.334, with a Cox–Snell R^2^ of 0.631 and a Nagelkerke R^2^ of 0.8450, reflecting substantial explained variance. The Hosmer–Lemeshow goodness-of-fit test demonstrated excellent model calibration (χ^2^(8) = 3.271, *p* = 0.916). Additionally, the database underwent a continuous quality review during both the data collection and processing phases to ensure data integrity, and to prevent the occurrence of missing values.

The inclusion of clinical and biochemical predictors provided a strong foundation for the analytic framework, with triglycerides, LDL-C, sdLDL-C, and VLDL emerging as the most consistent determinants of AD (Table 3A). When genetic variants were incorporated, the integrated model (Table 3B) yielded nearly equivalent performance, with a slightly reduced −2 log-likelihood of 217.026 and comparable Cox–Snell and Nagelkerke R^2^ values of 0.635 and 0.849, respectively. Model calibration remained strong, as indicated by a non-significant Hosmer–Lemeshow test (χ^2^(8) = 7.112, *p* = 0.525), thereby confirming the stability of associations across specifications. Collectively, these results demonstrate that lipid-related markers retain their central role as predictors of dyslipidemia, while the addition of *FTO* variants modestly enhances the explanatory power without altering the dominance of biochemical determinants.

Among the clinical and biochemical predictors evaluated in the multivariable models, triglycerides, LDL-C, sdLDL-C, and VLDL consistently emerged as the strongest independent determinants of AD, all retaining statistical significance with *p* < 0.05 (Table 3A). Triglycerides (OR = 2.825, 95% CI: 1.428–5.589, *p* = 0.003), LDL-C (OR = 7.920, 95% CI: 2.688–23.233, *p* < 0.001), sdLDL-C (OR = 2.825, 95% CI: 1.381–5.777, *p* = 0.004), and VLDL (OR = 2.776, 95% CI: 1.595–4.832, *p* < 0.001) demonstrated robust associations, underscoring their pivotal role in shaping the dyslipidemia profile.

In the integrated model including genetic variants (Table 3B), the predictive contribution of lipid fractions remained evident, with triglycerides (OR = 6.046, 95% CI: 2.374–15.396, *p* < 0.001), LDL-C (OR = 8.298, 95% CI: 2.425–28.390, *p* < 0.001), sdLDL-C (OR = 3.431, 95% CI: 1.328–8.867, *p* = 0.011), and VLDL (OR = 6.211, 95% CI: 2.685–14.371, *p* < 0.001) retaining strong significance. Notably, two *FTO* variants (rs9939609 and rs8050136) achieved independent significance, suggesting that genetic predisposition may act synergistically with biochemical parameters in modulating dyslipidemia risk.

Finally, the parsimonious specification (Table 3C) confirmed the stability of lipid-related predictors. Triglycerides (OR = 5.402, 95% CI: 2.281–12.793, *p* < 0.001), LDL-C (OR = 7.315, 95% CI: 2.280–23.467, *p* < 0.001), sdLDL-C (OR = 2.655, 95% CI: 1.159–6.086, *p* = 0.021), and VLDL (OR = 8.180, 95% CI: 3.726–17.958, *p* < 0.001) remained the most consistent determinants, reinforcing their role as core drivers of the atherogenic phenotype, independent of anthropometric measures and educational level, which did not retain statistical significance.

Multivariable logistic regression models of factors associated with AD: Odds ratios (OR) are presented with 95% confidence intervals (CIs). Models include (A) clinical predictors, (B) integrated clinical and genetic predictors, and (C) a parsimonious model with clinical variables only.

Analysis of *FTO* polymorphisms revealed that *rs9939609* and *rs8050136* were significantly associated with dyslipidemia, whereas *rs9940128* showed no independent effect. The A alleles of *rs9939609* and *rs8050136* were more frequent among cases and conferred a higher risk, findings that remained robust after adjustment for covariates and correction for multiple comparisons. To further explore these associations, we examined the genotypic and allelic distributions of the three *FTO* variants in cases and controls (Table 4). All genotypes in the control group conformed to Hardy–Weinberg equilibrium (*p* > 0.05), supporting the reliability of the observed frequencies. For *rs9939609*, the A allele was more frequent in cases than in controls. In the codominant model, carriers of the AA genotype exhibited nearly a fivefold higher risk compared with carriers of the TT genotype (OR = 4.977, 95% CI: 2.066–11.990, *p* < 0.001), an effect that persisted under the recessive model (OR = 2.459, 95% CI: 1.166–5.186, *p* = 0.018). These associations remained statistically significant after Bonferroni correction and adjustment for age, BMI, and educational level, indicating that the effect of *rs9939609* was independent of these covariates.

No significant associations were observed for *rs9940128* under any genetic model. Although GA and AA genotypes were more frequent in cases, the OR did not reach statistical significance, a finding likely explained by the low minor allele frequency (<10%) and limited statistical power for this variant. By contrast, rs8050136 showed strong associations. The frequency of the A allele was higher among cases than among controls, translating into a significant increase in risk. Under the codominant model, both CA (OR = 3.629, 95% CI: 1.650–7.982, *p* < 0.001) and AA genotypes (OR = 3.005, 95% CI: 1.317–6.860, *p* = 0.009) were significantly associated with dyslipidemia, with the CA genotype remaining significant after correction for multiple comparisons. Adjustment for clinical covariates did not attenuate these associations, reinforcing the independent contribution of rs8050136 to dyslipidemia risk.

Taken together, these findings demonstrate that *rs9939609* and *rs8050136* in the *FTO* gene are robustly associated with dyslipidemia in perimenopausal women, while *rs9940128* appears not to exert a major independent effect. The consistency of these associations, even after correction for multiple testing and adjustment for clinical factors, underscores the potential functional relevance of *FTO* variants in lipid metabolism and disease susceptibility.

### 2.3. Linkage Disequilibrium Analysis of FTO SNPs

**Figure 1:**
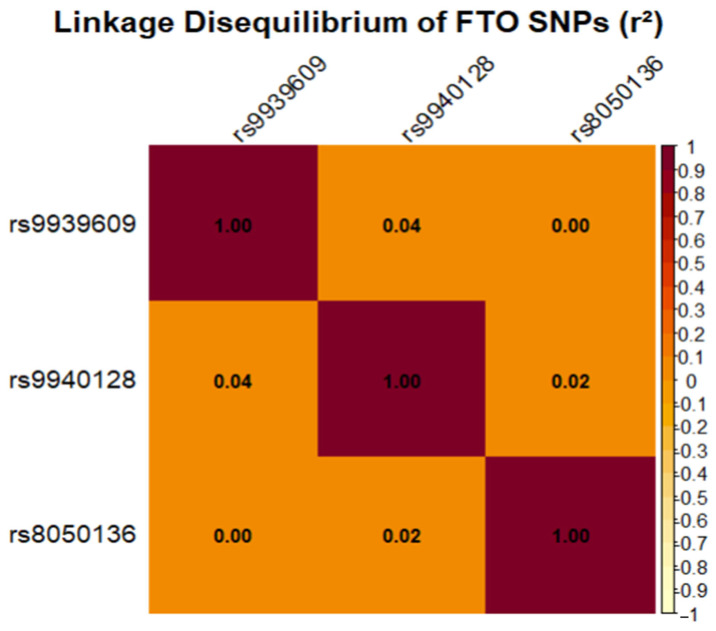
Linkage Disequilibrium Analysis of *FTO* SNPs. The heatmap depicts the r^2^ values among the three analyzed SNPs. Darker shades indicate stronger linkage disequilibrium. All values remained below 0.1, confirming the relative independence of the genetic variants under investigation.

To assess the extent of linkage disequilibrium among the *FTO* gene variants, an analysis was performed as illustrated in Figure 1. The results unequivocally demonstrated that the three SNPs segregated largely independently within this cohort. The squared correlation coefficients (r^2^) remained consistently low, not exceeding 0.05 for any marker pair (*rs9939609*–*rs9940128*, r^2^ = 0.045; *rs9939609*–*rs8050136*, r^2^ = 0.003).

These findings indicate that each polymorphism captures a distinct, non-redundant genetic signal. Importantly, this observation refines the haplotype analysis hypothesis: The associations observed with specific allele combinations are unlikely to result from inheritance within a haplotype block. Instead, they may reflect potential functional interactions or the additive contribution of independent genetic factors.

### 2.4. Individual Association of SNPs with AD

#### 2.4.1. Gene–Environment Interaction Model

The logistic regression analysis incorporating the interaction term between SNP rs8050136 and smoking status revealed significant associations (Table 5). Carriers of the AA genotype at rs8050136 exhibited an OR of 4.12 (95% CI: 1.49–11.95, *p* = 0.007), indicating a markedly increased AD risk. Smoking was also independently associated with an elevated AD risk (OR = 2.33, 95% CI: 1.03–5.44, *p* = 0.045).

#### 2.4.2. Distribution of Lipid Biomarkers by Genotype

**Figure 2:**
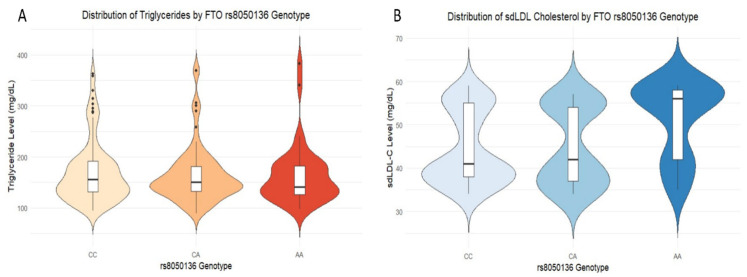
Distribution of lipid biomarkers by *FTO rs8050136* genotype. Within each violin plot, the central box indicates the median and interquartile range, while individual dots represent all data points; points falling far outside the main distribution are considered outliers. (**A**) Triglyceride distribution: Violin plots depict the density of triglyceride concentrations stratified by genotype. A trend toward higher values was evident among AA, accompanied by greater dispersion within this group. (**B**) sdLDL-C distribution: A similar pattern was observed for small dense low-density lipoprotein cholesterol, with the AA genotype exhibiting a distribution shifted toward higher concentrations compared with CC and CA genotypes.

The functional consequences of the *rs8050136* genotype on key lipid biomarkers were examined. The analysis revealed a clear and consistent dose–response effect of the risk allele “A” on the proatherogenic profile, as detailed in Figure 2. In Figure 2A, an upward gradient in triglyceride concentrations was observed across genotypes. Individuals with the CC genotype exhibited the lowest levels, heterozygous carriers (CA) displayed intermediate values, and homozygous risk carriers (AA) showed the highest median levels, thereby demonstrating a distinct additive effect of the “A” allele. A similar but even more pronounced pattern was replicated for small dense low-density lipoprotein cholesterol (sdLDL-C). As illustrated in Figure 2B, the distribution of sdLDL-C levels in the AA genotype was visibly shifted toward higher, more atherogenic concentrations compared with the CC and CA genotypes. These findings confirm that the risk conferred by the *rs8050136* polymorphism is not merely statistical but is expressed through an adverse quantitative phenotype, exerting a measurable influence on the core components of AD.

### 2.5. Haplotype Analysis of FTO

#### 2.5.1. Haplotype Frequencies

The haplotype analysis identified eight major combinations of the three *FTO* SNPs. Estimated haplotype frequencies in the study population ranged from 5.5% to 39.1%. The most common haplotype identified was T-G-C (39.1% frequency), which was subsequently used as the reference haplotype in the association models.

#### 2.5.2. Haplotype Association with AD

**Figure 3:**
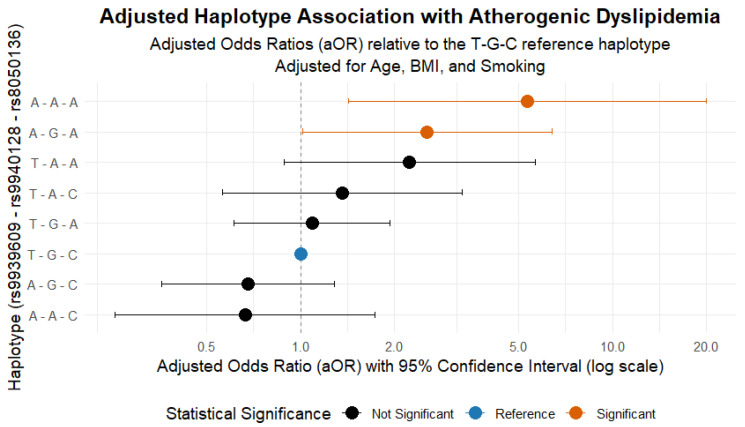
Adjusted forest plot of haplotype associations. The plot depicts the adjusted odds ratios (aOR) and 95% confidence intervals for the association of each *FTO* haplotype with AD, relative to the T-G-C reference haplotype. The model is adjusted for age, BMI, and smoking status. The vertical dotted line is set at an aOR of 1.0, representing the line of no effect; confidence intervals that cross this line are not statistically significant.

The evaluation of *FTO* haplotypes, adjusted for age, BMI, and smoking, revealed a marked specificity in the genetic risk architecture. As shown in Figure 3, the A-A-A haplotype (5.5% frequency) was associated with a substantially elevated AD risk (adjusted odds ratio [aOR] = 5.33; 95% CI: 1.42–20.00; *p* = 0.014). This finding confirms that the association is robust and independent of key clinical and lifestyle confounders.

Additionally, the A-G-A haplotype (5.6% frequency) was also found to confer a significant, though more moderate, risk (aOR = 2.54; 95% CI: 1.01–6.38; *p* = 0.048). No other haplotypes showed a statistically significant association.

These results are particularly insightful. First, they demonstrate that genetic risk is not uniformly distributed but is concentrated within specific, relatively infrequent allelic combinations. Second, the strong, supra-additive effect of the A-A-A haplotype (combining all three risk alleles) suggests a potential functional interaction among the variants. Consequently, these findings indicate that the accumulation of multiple risk alleles, even when not in strong linkage disequilibrium, can trigger an exponential increase in disease risk, providing a critical insight for genetic risk stratification.

## 3. Discussion

### 3.1. Sociodemographic and Clinical Characteristics

Analysis of the study cohort demonstrated that women with AD presented a markedly more unfavorable cardiometabolic profile than controls. This profile included significantly elevated concentrations of triglycerides, LDL-C, and sdLDL-C—the latter being a well-established marker of increased atherogenic risk. These findings confirm the usefulness of this phenotype as an intermediate marker of atherosclerotic disease and underscore its relevance as a framework for genetic research into cardiovascular risk. These observations are in line with emerging evidence that directly measured sdLDL-C as an independent predictor of atherosclerotic cardiovascular disease (ASCVD), even beyond the prognostic value of conventional lipid measures. For example, Schaefer et al. reported that elevated sdLDL-C (>50 mg/dL) is significantly associated with ASCVD risk across diverse populations [30].

In addition, recent findings suggest that the sdLDL-C to LDL-C ratio may further refine risk stratification: Yang et al. showed that higher quartiles of this ratio were associated with substantially higher odds of ASCVD, and that its predictive ability outperformed sdLDL-C alone [31]. Mechanistically, sdLDL particles are more susceptible to oxidation, more likely to penetrate the arterial intima, and have lower affinity for LDL receptors—characteristics that prolong their vascular residence and enhance atherogenic potential [3]. Noteworthily, in cohorts with apparently “optimal” LDL-C levels, elevated sdLDL-C still portends a higher coronary risk [32]. Taken together, these lines of evidence support the notion that, in profiles such as the one presented—with marked triglyceride, LDL-C, and sdLDL-C elevations—relying solely on traditional lipid markers may underestimate the true atherogenic burden. Therefore, the incorporation of sdLDL-C (or its ratio to LDL-C) into risk assessment algorithms could enable earlier and more targeted therapeutic intervention, especially for individuals with discordant lipid phenotypes.

In parallel with these atherogenic lipid abnormalities, the cardiometabolic burden was further aggravated by hemodynamic disturbances. The higher prevalence of hypertension and elevated systolic blood pressure among AD cases reinforces the concept that AD frequently coexists with hemodynamic imbalances that accelerate subclinical vascular injury. The coexistence of hypertension and dyslipidemia has been identified in multiple international studies as a particularly dangerous “cluster,” synergistically increasing cardiovascular risk by amplifying endothelial damage, arterial stiffness, and autonomic dysfunction [33]. This observation is particularly relevant because hypertension not only exerts mechanical stress on the arterial wall but also promotes endothelial dysfunction, oxidative stress, and vascular remodeling, thereby potentiating the deleterious impact of an adverse lipid profile.

Noteworthily, in this cohort, BMI and glycemia did not differ between cases and controls. This finding indicates that the observed atherogenic risk was not conditioned by overt obesity or classical glycemic alterations but rather by the intrinsic mechanisms of lipid metabolism. This observation supports the notion that AD can manifest in non-obese women and that cardiovascular risk is not invariably explained by adiposity. This finding aligns with growing evidence that AD can emerge independently of obesity or overt disturbances in glucose homeostasis. Several large-scale cohort studies have demonstrated that individuals with a normal BMI but adverse lipid phenotypes, often termed “metabolically unhealthy normal weight,” carry a disproportionately high cardiovascular risk [34]. In women, this phenotype may be particularly relevant, as hormonal fluctuations and sex-specific differences in lipid handling predispose them to increased concentrations of triglyceride-rich lipoproteins and small dense LDL, even in the absence of generalized adiposity [35]. Moreover, Mendelian randomization analyses have provided causal evidence linking genetically determined alterations in lipid metabolism to cardiovascular disease, independent of BMI or fasting glycemia, underscoring the primacy of lipid-driven mechanisms [36]. Importantly, this paradigm challenges the conventional reliance on BMI and glycemic indices as primary risk stratifiers, emphasizing the need to incorporate lipid subfractions, particle size, and functionality into cardiovascular risk assessment. In this context, women with ostensibly “normal” metabolic profiles may remain at a substantial residual risk, highlighting the clinical imperative for earlier detection and tailored preventive strategies.

An additional finding was the difference in marital status, with fewer AD cases reporting stable relationships. Although exploratory, this observation could reflect the impact of psychosocial factors on lipid metabolism. Recent studies have shown that chronic stress, social isolation, and a lack of social support are associated with systemic inflammation and metabolic dysfunction, contributing to cardiovascular disease progression [37]. These results suggest that a cardiometabolic risk assessment should encompass not only biological but also psychosocial determinants.

### 3.2. Multivariable Logistic Regression Model

The multivariable model confirmed that AD risk in this population was primarily determined by lipid fractions and blood pressure. LDL-C emerged as the strongest predictor (OR ≈ 8), corroborating its central role in atherogenesis, as reported in several large-scale cohorts [38]. Alongside LDL-C, triglycerides, sdLDL-C, and VLDL also displayed robust associations, forming a lipoprotein phenotype characterized by small, dense, and highly atherogenic particles. This profile mirrors the well-described “atherogenic triad”: hypertriglyceridemia, reduced HDL-C, and the predominance of sdLDL, a recognized predictor of coronary events.

In recent years, several prospective and cross-sectional investigations in female cohorts have corroborated the pathophysiologic relevance of a lipoprotein constellation dominated by elevated triglycerides, increased VLDL subclasses, and sdLDL particles. In the Women’s cohort of the Dallas Heart Study, for example, higher levels of triglyceride-rich lipoprotein cholesterol (TRL-C) and sdLDL cholesterol were independently associated with subclinical coronary atherosclerosis in women, even after adjusting for conventional risk factors [39]. Similarly, in a study of postmenopausal women, sdLDL-C levels were found to rise significantly independent of chronological aging, suggesting that menopausal status may amplify small dense LDL burden beyond general lipid shifts [40]. Moreover, analyses from the Women’s Ischemia Syndrome Evaluation cohort revealed that the triglyceride/HDL-C ratio—a surrogate of triglyceride enrichment and HDL depletion—was a stronger predictor of all-cause mortality and cardiovascular events than LDL-C in symptomatic women with ischemia, underscoring the incremental prognostic value of lipid particle composition over absolute LDL levels [41]. These lines of evidence reinforce that in women, the “atherogenic triad” phenotype not only reflects qualitative lipoprotein dysregulation but also carries independent predictive weight for cardiovascular outcomes beyond classical lipid metrics.

The associations of systolic and diastolic blood pressure highlight the synergistic interaction between dyslipidemia and hypertension. This coexistence accelerates subclinical atherosclerosis, increases arterial stiffness, and elevates cardiovascular event risk. In perimenopausal women, this interaction is particularly concerning, as it occurs during a period of declining estrogen protection, further compounding vascular vulnerability [14,42]. Interestingly, classical metabolic variables such as BMI and glycemia were not significant predictors in the model, suggesting that AD in this cohort may be driven by specific genetic and metabolic pathways rather than general adiposity [43]. This provides a clear pathophysiological framework for understanding the role of *FTO* polymorphisms, which appear to exert greater influence in the presence of this adverse lipoprotein profile.

### 3.3. Biological Mechanisms of the FTO Gene

This study confirms that the *FTO* rs8050136 polymorphism is significantly associated with AD in working perimenopausal women, independent of age and BMI. Homozygous carriers of the A allele exhibited more than a fourfold higher AD risk, highlighting this genotype as a potential biomarker of susceptibility.

Of particular importance is the observed gene–environment interaction with smoking. Tobacco exposure independently doubled AD risk, and in women carrying the AA genotype, the combined effect was even more pronounced. This finding is biologically plausible: smoking promotes oxidative stress, impairs HDL function, and increases the formation of sdLDL particles, thereby intensifying the atherogenic phenotype [44]. These results demonstrate the substantial amplification of genetic risk by environmental exposures and underscore the importance of targeted preventive interventions in genetically susceptible individuals.

Mechanistically, *FTO* variants are thought to modulate the expression of genes involved in adipogenesis, lipolysis, and insulin signaling, thereby influencing lipid metabolism beyond the effects of adiposity. The association of rs8050136 with hypertriglyceridemia and elevated sdLDL observed in this study reinforces this hypothesis, providing evidence of a quantitatively adverse metabolic phenotype.

### 3.4. Haplotype Associations with AD

Haplotype analysis, now adjusted for age, BMI, and smoking, revealed a marked specificity in the genetic risk architecture. The A-A-A combination emerged as the strongest predictor, conferring a more than fivefold increase in risk (aOR = 5.33; 95% CI: 1.42–20.00). Furthermore, the A-G-A haplotype also demonstrated a significant, though more moderate, association (aOR = 2.54; 95% CI: 1.01–6.38). The robustness of the A-A-A haplotype’s effect, even after adjusting for confounders, underscores its central role.

This supra-additive effect suggests that the accumulation of multiple risk alleles, even in the absence of strong linkage disequilibrium, exponentially increases the probability of developing the pathological phenotype. These findings are consistent with previous reports linking *FTO* pleiotropy to hepatic lipid metabolism and lipoprotein remodeling [20,45]. In Brazilian postmenopausal women, *rs8050136* was associated with higher lipid accumulation, suggesting that the adverse effects of this polymorphism may begin during perimenopause and intensify after menopause. Our results extend this evidence by showing that haplotypic combinations exert a significant risk even earlier, emphasizing the importance of haplotype-based genetic stratification in midlife women [26].

The finding that the A-A-A haplotype (aOR = 5.33) and the A-G-A haplotype (aOR = 2.54) exhibit an independent association with AD is noteworthy. However, it does not necessarily imply that individuals possessing these haplotypes should automatically be prescribed statin therapy, while those with other haplotypes can be disregarded. Genetic predispositions may influence lipid profiles and cardiovascular risk. Nonetheless, clinical decisions regarding statin therapy should be based on a comprehensive assessment of individual risk factors, including lipid levels, age, the presence of cardiovascular disease, and overall health status. The presence of a specific genetic variant alone is insufficient to dictate treatment protocols.

### 3.5. Clinical and Public Health Implications

These findings carry important implications for precision medicine. First, they demonstrate that *FTO* variants exert effects independent of obesity, interact with environmental exposures such as smoking, and manifest through both additive and supra-additive mechanisms. Second, they highlight the need to integrate genetic testing into cardiovascular risk assessment, particularly for perimenopausal women who face a convergence of occupational, hormonal, and genetic stressors.

From a public health perspective, the results underscore the need for prevention programs tailored to this population. Early identification of women carrying risk alleles, coupled with targeted lifestyle interventions—especially smoking cessation—could mitigate cardiometabolic risk. In Latin American contexts, where access to genetic testing remains limited, these findings provide an evidence base to prioritize resource allocation for vulnerable subgroups.

### 3.6. Integrating Clinical, Genetic, and Public Health Perspectives

Taken together, our findings delineate a coherent picture in which AD in working perimenopausal women is driven by a cluster of lipid abnormalities and hypertension, modulated by genetic susceptibility at the *FTO* locus, and exacerbated by environmental exposures such as smoking. Importantly, these results indicate that cardiovascular risk in this population is not explained by obesity alone but by qualitative alterations in lipoprotein fractions combined with genetic predisposition.

It was found that the A-A-A haplotype and the *rs8050136* SNP showed an independent association with AD, with ORs of 5.58. This finding is noteworthy and has been addressed in the Discussion; however, it does not imply that individuals carrying this haplotype should automatically receive statin therapy, nor that carriers of other haplotypes can be disregarded. Genetic predispositions, such as the A-A-A haplotype, may influence lipid profiles and cardiovascular risk. Nevertheless, clinical decisions regarding statin therapy should be based on a comprehensive assessment of individual risk factors, including lipid levels, age, the presence of cardiovascular disease, and overall health status. The presence of a specific genetic variant alone is insufficient to dictate treatment protocols. For instance, Urbano-Cano et al. [11] reported that the association between the FTO gene and an atherogenic lipid profile was not evident in patients already receiving statin therapy, suggesting that the effect of genetic variants on lipid profiles may be modulated by treatment. Therefore, although the A-A-A haplotype may be associated with AD, it should not be the sole determinant in prescribing statins; a personalized approach considering all relevant clinical factors is essential for effective management.

### 3.7. Strengths, Limitations, and Future Directions

The study’s strengths include the integration of detailed phenotyping, haplotype-based genetic analysis, and the explicit evaluation of gene–environment interactions. However, the study limitations that must be acknowledged are as follows: the sample size restricted statistical power for rarer haplotypes, and functional validation was not performed. Future research should employ multi-omics approaches to delineate the molecular pathways through which *FTO* variants influence lipid metabolism, and longitudinal designs are needed to assess whether these polymorphisms predict cardiovascular events over time.

In light of these considerations, extending research to include men and women without formal employment, as well as postmenopausal women, is essential for developing more comprehensive and equitable preventive health policies. Broadening participant representation will enable a more nuanced characterization of gene–environment interactions and help ensure that risk stratification frameworks are applicable across diverse population subgroups.

## 4. Materials and Methods

### 4.1. Study Design and Population

This case–control study included a total of 219 working perimenopausal women aged 40 to 55 years. Of these, 97 participants constituted the case group, having previously diagnosed dyslipidemia, while 122 comprised the control group, without any evidence of the condition. All participants were selected from the labor sector of the municipality of Popayán, Colombia.

### 4.2. Inclusion and Exclusion Criteria

Participants were included if they met the following criteria:

- Perimenopausal women, defined as those in the transitional stage toward menopause, exhibit irregular menstrual cycles according to established clinical criteria.
- Changes in cycle length
- The presence of vasomotor symptoms, including hot flashes and night sweats.
- Age between 40 and 55 years.
- Actively employed women.
- Provided informed consent for genetic, clinical, and paraclinical analyses.
- The exclusion criteria were as follows:
- Postmenopausal women (absence of menstruation for ≥12 consecutive months).
- Current pregnancy or lactation.
- Current or recent use (within the past 3 months) of medications affecting lipid or hormonal profiles (statins, fibrates, corticosteroids, hormone replacement therapy, hormonal contraceptives).
- History of ovarian surgery or hysterectomy.
- Participants with conditions causing significant systemic inflammation were excluded. This included autoimmune diseases (e.g., lupus, rheumatoid arthritis, multiple sclerosis, autoimmune thyroiditis), chronic inflammatory disorders (e.g., inflammatory bowel disease, severe psoriasis, psoriatic arthritis), and any active cancer.
- Non-acceptance of informed consent.

### 4.3. Data Collection

Demographic and clinical data: All participants were recruited after providing written informed consent according to the approved study protocol, and each volunteer was interviewed by a trained health professional to fill out a structured questionnaire to establish socio-demographic characteristics (age and occupational status), personal clinical history (dyslipidemia, hypertension, diabetes, body mass index (BMI)), biochemical data (total cholesterol, triglycerides, HDL, VLDL, LDL, sdLDL-C), and smoking habits (never, former, current). All of the questionnaires, procedures, and protocols were reviewed and approved by the ethics committee of the University of Cauca, and the guidelines used in the review were based on the bioethical principles established in the Helsinki Declaration of 2024 and the parameters outlined in Resolution 8430 of the Colombian Ministry of Health in 1993. Female participants were enrolled in the project “Social Determinants of Metabolic Syndrome in a Working Population in the Municipality of Popayan” (VRI-5694) over a one-year period.

### 4.4. Cardiovascular Risk Factor Measurements

Cardiovascular risk factors were assessed by measuring each patient’s height, weight, and resting blood pressure during the examination. Hypertension was defined as a systolic blood pressure ≥ 140 mm Hg and/or a diastolic blood pressure ≥ 90 mm Hg [46]. The calculation of BMI involved dividing weight by height squared (kg/m2). The subjects were split into three weight groups: normal weight (BMI 18.5 < 25.0), overweight (BMI 25.0 < 30.0), and obese (BMI ≥ 30) [47]. Blood samples were obtained for biochemical analysis, and medical records were examined for clinical diagnosis, in order to confirm the existence of personal risk factors. The lipid phenotype characteristic of AD was defined by the coexistence of three abnormalities: elevated triglycerides (≥150 mg/dL), reduced high-density lipoprotein cholesterol (<50 mg/dL in women). The LDL-c values considered “within the normal range” were specified and defined as LDL-c < 130 mg/dL, while an increased concentration of small dense LDL cholesterol (sdLDL-C) was considered above 50.0 mg/dL. The applied cutoff values were based on recommendations from the Colombian consensus for the diagnosis and management of dyslipidemia in adults, as well as the 2019 ESC/EAS Guidelines for the management of dyslipidemias: lipid modification to reduce cardiovascular risk [2].

### 4.5. Genetic Data

Blood samples were collected from all participants, and DNA was extracted using the salting-out method [48].

*FTO* gene polymorphisms were genotyped using Tetra-Primer ARMS PCR (Amplification Refractory Mutation System) for the determination of the *rs9939609* (T > A) variant. For the *FTO* polymorphisms *rs9940128* (G > A) and *rs8050136* (C > A), PCR-RFLP (Polymerase Chain Reaction–Restriction Fragment Length Polymorphism) techniques were employed using the restriction enzymes *MspI* and *MseI*, respectively (New England Biolabs) (Table 6). The genomic regions containing the polymorphisms of interest were amplified via PCR using a thermocycler (BioRad, Popayán, Colombia), following the manufacturer’s instructions. Genotypes were visualized on a 3% agarose gel stained with ethidium bromide.

### 4.6. Statistical Analysis and Data Visualization

In this study, data were obtained from three sources, including sociodemographic, environmental, and genetic variables, following the survey structure. All variables were stored in a database using SPSS for Windows, version 26 (SPSS Inc., Chicago, IL, USA) (accessed June 2025). The normality of quantitative variables was initially assessed using the Kolmogorov–Smirnov test with Lilliefors significance correction. Quantitative variables that did not meet the assumption of normality were described using the median and interquartile range due to their non-parametric distribution, while categorical variables were summarized as frequencies and percentages. Chi-square tests were used to compare proportions. For continuous variables, comparisons between two groups were performed using the Mann–Whitney U test, and comparisons across more than two groups were conducted using the Kruskal–Wallis test. A *p*-value of <0.05 was considered statistically significant. To evaluate the association between each variable and susceptibility to AD, the dichotomous outcome variable (case vs. control) was regressed against each biological factor to estimate the OR and 95% confidence intervals using a multivariable logistic regression model. ORs were adjusted for age and place of origin. The chi-square statistic generated by the regression model tested the null hypothesis of no association between the subject’s status and each variable. All *p*-values correspond to two-tailed tests, with a significance threshold set at <0.05. Allelic and genotypic frequencies, as well as Hardy–Weinberg equilibrium, were calculated using PLINK version 1.9 (PLINK: Whole genome data analysis toolset accessed on 9 November 2025). Significant deviation was defined as *p* < 0.001, a threshold adopted to minimize type I errors in genetic association studies. Additionally, minor allele frequency (MAF) and genotyping call rates were calculated specifically for twehe *FTO* gene polymorphisms. For the association analysis between *FTO* genotypes and AD, codominant, dominant, and recessive genetic models were applied using unconditional logistic regression. ORs were calculated with 95% confidence intervals (95% CIs). To account for multiple testing (three SNPs × three genetic models = nine comparisons), Bonferroni correction was applied, setting the significance threshold at α = 0.05/9 = 0.0055. For the logistic regression models, the overall fit was evaluated using the −2 log-likelihood and the Hosmer–Lemeshow goodness-of-fit test, and explanatory power was assessed using the Cox–Snell R2 and Nagelkerke R2. Additionally, the variance inflation factor (VIF) was calculated for all predictors included in the regression models to assess potential multicollinearity.

The association between genetic variants and AD was assessed using multivariable logistic regression models to estimate ORs with their corresponding 95% confidence intervals (CIs). All models were adjusted for potential confounders, including age and body mass index (BMI). The gene–environment interaction was explicitly evaluated by incorporating a multiplicative term between SNP genotype and smoking status.

Linkage disequilibrium (LD) among the SNPs was quantified using the squared correlation coefficient (r^2^) and visualized with a heatmap. Haplotype analysis was conducted to examine the combined effect of alleles; haplotype frequencies were estimated, and their associations with disease were tested using the haplo.glm function, adjusting for age, BMI, and smoking status as covariates.

A *p*-value of <0.05 was considered statistically significant for all analyses. The main R libraries employed for data analysis and visualization included tidyverse, readxl, haplo.stats, and corrplot (R version 4.4.3).

## 5. Conclusions

This study identifies a significant but not exclusive contribution of *FTO* gene variants—particularly rs8050136 and specific haplotypes (A–A–A and A–G–A)—to the risk of atherogenic dyslipidemia (AD) in working perimenopausal women. The observed associations remained independent of obesity indices and were amplified by environmental exposures such as smoking, suggesting a complex gene–environment interplay influencing lipid metabolism.

However, the overall explanatory power of the models indicates that lipid fractions (LDL-C, triglycerides, sdLDL-C, and VLDL) remain the predominant determinants of AD, while *FTO* polymorphisms exert a modest additive effect rather than a central pathogenic role. These findings therefore support a model in which *FTO* genetic variability contributes to susceptibility through the modulation of lipid handling pathways, but within a multifactorial network dominated by biochemical and lifestyle factors.

The study results emphasize the importance of integrating genetic, clinical, and environmental parameters in precision cardiovascular risk assessments, rather than attributing causality to *FTO* variants alone. Further studies employing functional genomics and longitudinal designs are warranted to clarify the molecular mechanisms linking *FTO*-mediated epitranscriptomic regulation to lipid metabolism and to determine their predictive value for cardiovascular outcomes across diverse populations.

## Figures and Tables

**Table 1:** Demographic and clinical characteristics of the study population.

Variable	Case	Control	Total (*n* = 219)	*p*-Value
Age (Years) (Median ± IQR)	46.4 ± 4.75	47.4 ± 4.62	48 ± 13	0.175
Systolic Pressure (mmHg) (Median ± IQR)	143 [120–170]	122 [110–140]	136 [120–140]	0.011
Diastolic Pressure (mmHg) (Median ± IQR)	92 [70–99]	87 [70–90]	90 [70–100]	0.060
Glycemia (mmHg) (Median ± IQR)	103 [89–115]	101 [87–105]	102 [88–108]	0.247
Cholesterol (mg/dL) (Median ± IQR)	206 [168–236]	200 [177–218]	203 [176–224]	0.719
HDL (mg/dL) (Median ± IQR)	44 [38–51]	47 [37–53]	45 [38–52]	0.158
Triglycerides (mg/dL) (Median ± IQR)	172 [140–177]	134 [105–150]	151 [120–170]	<0.001
LDL (mg/dL) IQR	138 [117–150]	118 [99–115]	127 [107–150]	<0.001
sdLDL-C (mg/dL) (Median ± IQR)	42 [32–55]	28 [20–30]	35 [30–40]	<0.001
VLDL (mg/dL)	33 [24–40]	31 [24–35]	32 [24–40]	0.331
BMI (kg/m^2^) (Median ± IQR)	26 [22–28]	25 [22–28]	27 [23–29]	0.065
Waist circumference (Median ± IQR)	91 [83–99]	91 [83–97]	91 [83–90]	0.804
Body fat measurement (Median ± IQR)	32 [23–40]	31 [22–38]	33 [24–41]	0.121
Hypertension (*n*, %)	Yes	Yes	163 (74.4%)	0.034
	79 (81.4%)	84 (68.9%)		
	No	No	56 (25.6%)	
	18 (18.6%)	38 (31.1%)		
Diabetes (*n*, %)	Yes	Yes	67 (30.6%)	0.493
	32 (33.0%)	35 (28.7%)		
	No	No	152 (69.5%)	
	67 (67.0%)	87 (71.3%)		
Area of Origin (*n*, %)	Urban:	Urban:	182 (83.1%)	0.709
	80 (82.5%)	102 (83.6%)		
	Rural:	Rural:	37 (16.9%)	
	17 (17.5%)	20 (16.4%)		
In a Relationship (*n*, %)	Yes	Yes	104 (47.5%)	<0.001
	38 (39.2%)	77 (63.1%)		
	No	No	115 (52.5%)	
	59 (60.8%)	45 (36.9%)		
Educational Level (*n*, %)	Elementary school	Elementary school	123 (56.2%)	0.162
	62 (64.0%)	61 (50.0%)		
	Middle School/High School	Middle School/High School	59 (26.9%)	
	22 (22.7%)	37 (30.3%)		
	Technical education	Technical education	16 (7.3%)	
	7 (7.2%)	9 (7.4%)		
	College/University	College/University	21 (9.6%)	
	6 (6.2%)	15 (12.3%)		
Income (*n*, %)	<1 SMLV	<1 SMLV	82 (37.4%)	0.298
	40 (41.2%)	42 (34.4%)		
	1 SMLV	1 SMLV	64 (29.2%)	
	30 (30.9%)	34 (27.9%)		
	>1 SMLV	>1 SMLV	73 (33.3%)	
	27 (27.8%)	46 (37.7%)		
Smokers (*n*, %)	Yes	Yes	95 (43.4%)	0.104
	48 (49.5%)	47 (38.5%)		
	No	No	124 (56.6%)	
	49 (50.5%)	75 (61.5%)		

Clinical, biochemical, and sociodemographic characteristics of cases and controls. Data are presented as median [IQR] for most continuous variables and as frequency (percentage) for categorical variables. Age is presented as Mean ± SD. Comparisons between groups were performed using the Mann–Whitney U test for continuous variables and the chi-squared test for categorical variables.

**Table 2:** Selected risk factors and OR (95% CIs) for AD.

Variable	OR	95% CI	*p*-Value
Systolic Pressure (mmHg)	2.59	1.286–5.254	0.008
Diastolic Pressure (mmHg)	4.57	2.575–8.112	<0.001
Glycemia (mg/dL)	1.78	0.819–3.878	0.145
Cholesterol (mg/dL)	1.00	0.997–1.012	0.249
HDL (mg/dL)	1.01	0.993–1.040	0.169
Triglycerides (mg/dL)	3.14	1.802–5.471	<0.001
LDL (mg/dL)	2.15	1.247–3.701	0.006
sdLDL-C (mg/dL)	2.63	1.505–4.602	<0.001
VLDL (mg/dL)	2.57	1.484–4.467	<0.001
Educational Level	1.71	1.027–3.057	0.040
BMI (kg/m^2^)	1.84	0.915–3.735	0.087
*FTO*_rs9939609 *T > A*	2.987	1.496–5.558	0.002
*FTO*_rs9940128 *G > A*	1.938	0.807–4.657	0.139
*FTO*_rs8050136 C > A	3.629	1.650–7.982	<0.001
Percentage Body Fat	1.016	0.994–1.039	0.164
Body Mass Index (kg/m^2^)	1.051	0.994–1.111	0.082
Waist Circumference Measurement	1.001	0.981–1.022	0.927

Abbreviations: OR, odds ratio; CI, confidence interval.

**Table 3:** (**A**) Multivariable logistic regression model of factors associated with AD. (**B**) Integrated multivariable model with clinical and genetic predictors. (**C**) Parsimonious multivariable logistic regression model of clinical predictors.

(**A**)					
Variables	Standard Error	OR	Wald	95%CI (Lower–Upper)	*p*-value
Systolic Pressure (mmHg)	0.283	1.835	4.618	1.055–3.193	0.032
Diastolic Pressure (mmHg)	0.289	2.060	6249	1.169–3.629	0.012
Glycemia (mg/dL)	0.294	0.817	0.470	0.459–1.455	0.493
Cholesterol (mg/dL)	0.004	1.004	1.330	0.997–1.012	0.249
HDL (mg/dL)	0.012	1.016	1.890	0.993–1.040	0.169
Triglycerides (mg/dL)	0.348	2.825	8.906	1.428–5.589	0.003
LDL (mg/dL)	0.551	7.92	14.087	2.688–23.233	<0.001
sdLDL-C (mg/dL)	0.365	2.825	8.095	1.381–5.777	0.004
VLDL (mg/dL)	0.283	2.776	13.032	1.595–4.832	<0.001
Educational Level	0.385	1.422	0.835	0.668–3.024	0.361
BMI (kg/m^2^)	0.029	1.051	3.031	0.994–1.111	0.082
(**B**)					
Predictor		OR		95% CI	*p*-value
Total Cholesterol (mg/dL)		1.409		0.643- 3.086	0.391
HDL-C (mg/dL)		1.022		0.990–1.054	0.186
Triglycerides (mg/dL)		6.046		2.374–15.396	<0.001
LDL (mg/dL)		8.298		2.425–28.390	<0.001
sdLDL-C (mg/dL)		3.431		1.328–8.867	0.011
VLDL (mg/dL)		6.211		2.685–14.371	<0.001
FTO_rs9939609 T > A		4.062		1.727–9556	<0.001
FTO_rs9940128 G > A		1.687		0.545–5.219	0.364
FTO_rs8050136 C > A		3.741		1.454–9623	0.006
(**C**)					
Predictor		OR		95% CI	*p*-value
HDL-C (mg/dL)		1.025		0.995–1.056	0.109
Triglycerides (mg/dL)		5.402		2.281–12.793	<0.001
LDL (mg/dL)		7.315		2.280–23.467	<0.001
sdLDL-C (mg/dL)		2.655		1.159–6.086	0.021
VLDL (mg/dL)		8.180		3.726–17.958	<0.001
Percentage Body Fat		0.974		0.931–1.018	0.245
Body Mass Index (kg/m^2^)		1.118		0.994–1.257	0.063
Waist Circumference Measurement		0.989		0.958–1.021	0.490
Educational Level (Sec./Tech. vs. Prof.)		1.348		0.690–2.633	0.383

**Table 4:** Genotype and allelic frequencies of *FTO rs9939609 T > A*, *FTO_rs9940128 G > A*, and *FTO_rs8050136 C > A* variants in cases of perimenopausal working women and controls.

*FTO* Polymorphism	Case *n* (%)	Control *n* (%)	OR (95% CI)	*p*-Value
rs9939609 T > A codominant				
TT	58 (59.8)	59 (48.4)	1.0	-
TA	17 (17.5)	50 (41.0)	1.721(0.793–3.739)	0.170
AA	22 (22.7)	13 (10.7)	4.977(2.066–11.990)	<0.001
rs9939609 T > A Dominant				
TT	58 (59.8)	59 (48.4)	1.0	-
TA + AA	39 (40.2)	63 (51.6)	1.588(0.926–2.723)	0.093
rs9939609 T > A Recessive				
TT + TA	75 (77.31)	109 (89.3)	1.0	-
AA	22 (22.7)	13 (10.7)	2.459(1.166–5.186)	0.018
rs9940128 G > A codominant				
GG	50 (51.5)	82(67.2)	1.0	-
GA	34 (35.1)	29(23.8)	1.938 (0.807–4.657)	0.139
AA	13 (13.4)	11(9.0)	1.008 (0.392–2.590)	0.987
rs9940128 G > A Dominant				
GG	50 (51.5)	82(67.2)	1.0	
GG + GA	47 (48.5)	40(32.8)	1.733 (0.907–3.657)	0.119
rs9940128 G > A Recessive				
GG + GA	84 (86.6)	111(91.0)	1.0	-
AA	13 (13.4)	11 (9.0)	0.640 (0.273–1.500)	0.305
rs8050136 C > A codominant				
CC	40 (41.2)	67 (54.9)	1.0	-
CA	31 (32.0)	43 (35.2)	3.629 (1.650–7.982)	*p* < 0.001
AA	26 (26.8)	12 (9.8)	3.005 (1.317–6.860)	0.009
rs8050136 C > A Dominant				
CC	40 (41.2)	67 (54.9)	1.0	
CA + AA	57 (58.8)	55 (45.1)	0.936 (0.967–2.976)	0.052
rs8050136 C > A Recessive				
CC + CA	71 (73.2)	110 (90.2)	1.0	
AA	26 (26.8)	12 (9.8)	1.562(0.667–3.659)	0.305

Abbreviations: OR, odds ratio; CI, confidence interval. The “-” appearing in the *p*-value column for the reference genotype indicates that the *p*-value is not applicable in this case.

**Table 5:** Logistic regression analysis: SNP rs8050136 × Smoking Interaction.

Variable	OR	95% CI	*p*-Value
Systolic Pressure	1.98	0.85–4.75	0.116
Diastolic Pressure	4.12	1.49–11.95	0.007
Smoking	2.33	1.03–5.44	0.045
Age	0.95	0.89–1.01	0.109
BMI	0.96	0.90–1.02	0.192
Interaction CA × Smoking	0.41	0.11–1.50	0.183
Interaction AA × Smoking	1.44	0.24–12.34	0.706

OR: odds ratio calculated relative to the reference haplotype T-G-C; CI: confidence interval.

**Table 6:** The primers used for the detection of *FTO* gene polymorphism.

Polymorphism	Primers (5′→3′)	Genotyping Method	Restriction Enzyme
*rs9939609* (T > A)	External F: gttctacagttccagtcatttttgacagc External R: agcctctctaccatcttatgtccaaaca Internal F(A):000 taggtccttgcgactgctgtgaatata Internal R(T): gagtaacagagactatccaagtgcatctca	Tetra-Primer ARMS PCR	N/A
*rs9940128* (G > A)	F: 5′AGGCCTCAGCTTCCCTGAACTGG3′ R: 5′TGCCATGGAAAATCTGGCTCATGGT3′	PCR-RFLP	*MspI*
*rs8050136* (C > A)	F: ATGCCAGTTGCCCACTGTGGGCATT R: GCAAAATTTCACACACCCAAGATGGTCCATG	PCR-RFLP	*MseI*

## Data Availability

The original contributions presented in this study are included in the article. Further inquiries can be directed to the corresponding author.
